# Pollen viability-based heat susceptibility index (HSIpv): A useful selection criterion for heat-tolerant genotypes in wheat

**DOI:** 10.3389/fpls.2022.1064569

**Published:** 2022-12-01

**Authors:** Irum Khan, Jiajie Wu, Muhammad Sajjad

**Affiliations:** ^1^ Department of Biosciences, COMSATS University Islamabad, Islamabad, Pakistan; ^2^ State Key Laboratory of Crop Biology, Shandong Agricultural University, Tai’an, Shandong, China

**Keywords:** wheat, heat stress, pollen viability, heritability, genetic advance

## Abstract

Terminal heat stress during reproductive stage in wheat (*Triticum aestivum* L.) causes pollen grain sterility and has a drastic impact on wheat crop production. Finding genotypes with high pollen viability under heat stress is crucial to cope with the impact of climate change through developing heat-tolerant cultivars. To assess the effect of terminal heat stress on pollen viability in a panel of spring wheat genotypes (*N* = 200), RCBD (randomized complete block design) field trials were conducted under normal and heat stress conditions for two consecutive years (2020–2021 and 2021–2022). Analysis of variance showed significant variation in genotypes, treatments, and genotype × treatment interaction. Fifty and 46 genotypes were categorized as heat tolerant (*HSI*_*pv*_ < 0.5) in the first and second year, respectively. Twelve genotypes, namely, Chenab-70, Pari-73, Pak-81, MH-21, Punjab-76, NIFA-Aman, NUWYT-63, Swabi-1, Nisnan-21, Frontana, Amin-2000, and Pirsabak-2004, were found to be heat tolerant across the years. The violin plot displayed a trend of improvement in heat tolerance (*HSI*_*pv*_ < 0.5) over the period of time in many modern wheat varieties. However, some modern wheat varieties released after 2001 such as Janbaz-09 (57%), Ghazi-2019 (57%), and Sindhu-16 (43%) had very low pollen viability under heat stress conditions. The results of phenotypic coefficient of variance (PCV%), genotypic coefficient of variance (GCV%), broad sense heritability (h^2^_bs_), and genetic advance (GA) suggested the major contribution of genetic factors in controlling pollen viability trait. Higher values of h^2^_bs_ and GA under heat stress conditions suggested pollen viability as a heat tolerance trait controlled by additive genetic effects. Taken together, these results suggested pollen viability as a useful trait for selection in early generations under elevated temperatures. The genotypes identified as heat tolerant in both years can be used as genetic resources for breeding cultivars with higher pollen viability under elevated temperature conditions.

## Introduction

Global production of major staple food crops like wheat, rice, and maize is at risk due to climate change. The negative impact of climate change on crop production is predicted to threaten global food security (Sharma et al., 2019). The IPCC (Intergovernmental Panel on Climate Change) forecasts an increase of 1–1.5°C in annual daily maximum temperature between 2030 and 2052 (IPCC, 2018). Reproductive stages are more prone to high-temperature stress in cereals (Nasehzadeh and Ellis, 2017; Mamrutha et al., 2020) and an increase of a few degrees above the optimum temperature during pollen grain development lowers seed setting due to pollen infertility (Bita and Gerats, 2013; Bokszczanin, 2013).

Abnormal pollen development due to heat stress has been widely observed in many crops causing incomplete male sterility and consequently lower grain yield (Sakata and Higashitani, 2008; De Storme and Geelen, 2014). The reproductive success of a crop variety under heat stress mainly depends on pollen production, pollen viability, and seed set. A strong correlation between pollen viability and seed set has been reported (Saini and Aspinall, 1982; Saini et al., 1983; Prasad et al., 2006; Masthigowda et al., 2022).

Wheat (*Triticum aestivum* L) is a leading staple crop grown in 89 countries with the largest harvested area (219 million ha) and the second largest production (760.9 million tons) among cereals (FAO, 2020, www.fao.org). The grain yield of wheat crop depends on seed setting, which, in turn, relies on reproductive development and successful fertilization (Reynolds et al., 2012). Heat stress in wheat reduces pollen viability, thus causing poor fertilization and seed setting (Saini and Aspinall, 1982; Ferris et al., 1998; Akter and Rafiqul Islam, 2017). Wheat is a heat-sensitive crop (Akter and Rafiqul Islam, 2017) and is susceptible to high temperature during vegetative and reproductive stages (Balla et al., 2019). The optimum temperature for spring wheat during anthesis and grain filling stages ranges from 12 to 22°C (Farooq et al., 2011). The heat stress of 30°C or higher temperature for three consecutive days, during pollen formation, significantly decreased grain setting and yield in wheat (Saini and Aspinall, 1982). The degradation of the tapetum tissues due to heat stress during microspore meiosis resulted in pollen sterility (Saini et al., 1984). All published reports concluded that heat stress at or just before anthesis caused pollen sterility and affected seed set in wheat (Saini and Aspinall, 1982; Ferris et al., 1998; Prasad et al., 2006; De Storme and Geelen, 2014; Masthigowda et al., 2022; Mamrutha et al., 2022). Therefore, identifying wheat genotypes with high pollen viability under heat stress is prerequisite for breeding heat-tolerant cultivars (Gulnaz et al., 2019; Impe et al., 2020). Substantial genetic variability has been reported for pollen viability under heat stress in wheat; however, these studies used few genotypes (Prasad et al., 2006; Narayanan et al., 2016; Okechukwu et al., 2016). Bheemanahalli et al. (2019) used 28 diverse spring wheat genotypes, but the experiment was performed under controlled environment conditions. The response of genotypes under controlled lab conditions is hardly extrapolated to field conditions. Recently, only 10 spring wheat genotypes were used to study the effect of heat stress under field conditions for two crop seasons (Masthigowda et al., 2022). In this field experiment, strong correlation of pollen viability with grain weight and grain numbers was observed under heat stress condition. All available studies on pollen viability documented its strong correlation with yield traits under heat stress. Herein, we performed a field experiment for two consecutive years under normal and heat stress conditions using 200 spring wheat genotypes. Pollen viability-based heat susceptibility index ( *HSI*_*pv*_ ) was calculated and suggested as a useful selection criterion for heat-tolerant wheat lines to breed future heat-tolerant cultivars. Phenotypic coefficient of variance (PCV%), genotypic coefficient of variance (GCV%), broad sense heritability (h^2^bs), and genetic advance (GA) were estimated to determine the genetic nature of pollen viability trait under normal and heat stress conditions. The heat-tolerant wheat genotypes identified in this study can be used as potential parents for breeding heat-tolerant wheat cultivars.

## Materials and methods

### Plant material

The pure seed of 200 spring wheat genotypes including land races, pre-green revolution, post-green revolution, and recent cultivars and advanced lines were collected and arranged for three replications.

### Field experiments

The experiment was conducted at the National Agricultural Research Center (NARC), Islamabad, Pakistan, for two growing seasons (2020–2021 and 2021–2022). The sowing dates for the period 2020–2021 and 2021–2022 were November 15, 2020 and November 13, 2021, respectively. The seeds of about 200 diverse spring wheat genotypes were grown in randomized complete block design (RCBD) with three replications. The seeds were sown with wheat planter in 1.2 m × 3 m plots, consisting of six rows, 20 cm apart. The standard agronomic practices were used in each experiment to raise good quality crops. The crop was subjected to terminal heat stress before spike initiation stage by covering one set with a transparent plastic sheet. Openings were built at regular intervals throughout the enclosure to enable free movement of air, in order to prevent relative humidity buildup inside the enclosure. An increase of 1–3°C was recorded at different times during heat stress period. Another set not covered was used as control.

### Pollen viability test

Three central spikelets per replication were collected from all 200 spring wheat genotypes during the third weeks of February 2021 and February 2022. The maximum temperature ranges observed during the third weeks of February 2021 and February 2022 were 23–26°C and 20–23°C, respectively (https://www.accuweather.com). Anthers were removed with a sharp needle and stored at 4°C in a refrigerator for microscopic study of pollen viability. Modified ALEXANDER test (Dafni and Firmage, 2000) was used for pollen viability test. The stained pollen grains were recorded under a compound microscope (Olympus). The darkly stained pollens were fertile and lightly stained pollens were sterile. Pollen viability was counted as the ratio of number of stained pollen to total number of pollen grains and quantified as percentage (Prasad et al., 2006).

### Statistical analysis

#### Pollen viability-based heat susceptibility index, *HSI*_*pv*_

The pollen viability-based heat susceptibility index **(** *HSI*_*pv*_ ) was calculated in Microsoft Excel 2016 using percent pollen viability values of terminal heat stress and normal conditions following the formula below (Fischer and Maurer, 1978). Based on, HSI_pv_ genotypes were grouped into three classes, tolerant (*HSI*_*pv*_ <0.5), moderately tolerant ( *HSI*_*pv*_ 0.5–0.99), and susceptible (HSI_pv_ > 1.0).


$$HSI_{pv}=1-\left(\frac{X_{stress}}{X_{normal}}\right)/1-\left(\frac{{\bar{X}}_{stress}}{{\bar{X}}_{normal}}\right)$$

#### Descriptive statistics, analysis of variance, and scatter plot

Descriptive statistics, analysis of variance (ANOVA), and scatter plot were calculated using Jamovi 2.3.18 (R Core Team, 2021; The jamovi project, 2022). Descriptive statistics were calculated using percent pollen viability. The scatter plot was constructed using *HSI*_*pv*_ values of the two crops’ seasons on *y*-axes and percent pollen viability of genotypes on *x*-axes. To show the trend of *HSI*_*pv*_ over the period of time, a violin plot was made using Jamovi 2.3.18 (R Core Team, 2021; The jamovi project, 2022). The panel of 200 genotypes was categorized in pre-green revolution, post-green revolution, and modern wheat varieties following Gohar et al. (2022).

#### Phenotypic coefficient of variance, genotypic coefficient of variance, broad sense heritability, and genetic advance

The basic genetic parameters—PCV%, GCV%, h^2^bs, and GA—were calculated using percent pollen viability values under normal and stress conditions. The following formulas (Allard, 1960) were used in Microsoft Excel 2016 to calculate these parameters.

$\text{i. PCV }\left(\%\right)=\frac{\sqrt{2^{\text{p}}}}{\bar{X}}\;\times 100$

$\text{ii. GCV }\left(\%\right)=\frac{\sqrt{2^{\text{g}}}}{\bar{X}}\;\times 100$

${\text{iii. h}}^{2}{\text{b}}_{\text{s}}\left(\%\right)=\frac{\sigma^{2}\text{g}}{\sigma^{2}\text{p}}\;\times 100$

$\text{iv. GA}=\text{k}\times \sqrt{\sigma^{2}\text{p}}\times {\text{h}}^{2}{\text{b}}_{\text{s}}$

  where

  σ^2^p = phenotypic variance

  σ^2^g = genotypic variance

$\;\bar{X}$ = mean value

  K-constant = 2.06 at 5% selection intensity

## Results

**Figure 1:**
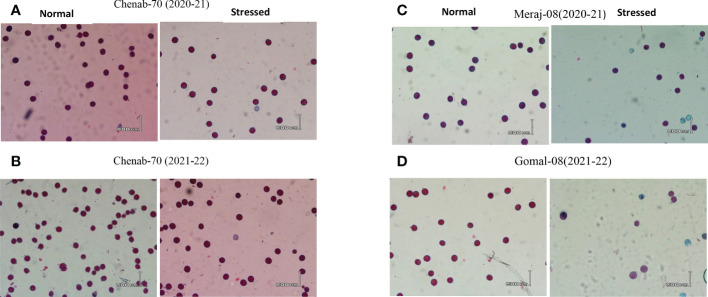
Microphotographs (10×) of wheat high-tolerant lines **(A, B)** during 2020–2021 and 2021–2022 under control and heat stress conditions. **(C, D)** Susceptible wheat lines during 2020–2021 and 2021–2022 under control and heat stress conditions.

The pollen viability data of 200 spring wheat lines grown under normal and terminal heat stress conditions were recorded for two crop periods, 2020–2021 and 2021–2022 (**Tables S1, S2**). The descriptive statistical parameters for all 200 genotypes and for the pre-green revolution, post-green revolution, and modern wheat varieties were calculated (**Tables S1–S5**). Pollen viability was determined as percentage of darkly stained pollens. Heat stress affected pollen viability in both years due to sensitivity of reproductive stage ~30°C temperature. During 2020–2021 under normal conditions, 100% pollen viability was recorded in 5 genotypes, 90%–99% was recorded in 184 genotypes, 80%–89% was recorded in 10 genotypes, and the minimum pollen viability (76%) was observed in commercial variety “Sindhu-16” released in 2016. Under heat stress conditions, 90%–97% pollen viability was observed in 134 genotypes, 80%–89% was observed in 49 genotypes, and 71-79% was observed in 14 genotypes. Low pollen viability was recorded in some wheat varieties—Meraj-08 (66%), Gulzar-19 (66%), and Ujala-15 (62%) (**Table S1** and **Figure 1**).

During 2021–2022 under normal conditions, 100% pollen viability was observed only in “Gomal-08”, 90%–99% was observed in 165 genotypes, and 81%–89% was observed in 32 genotypes. Low pollen viability was observed in two old varieties, Chakwal-86 (76%) and Punjab-96 (78%). Under heat stress conditions, 90%–98% pollen viability was observed in 81 genotypes, 80%–89% was observed in 76 genotypes, 70%–78% was observed in 25 genotypes, and 60%–67% was observed in 14 genotypes. Some wheat varieties including Janbaz-09 (57%), Ghazi-2019 (57%), Sindhu-16 (43%), and Manthar-2003 (0%), released after 2001, had very low pollen viability under heat stress conditions during the period 2021–2022 (**Table S1** and **Figure 1**).

**Figure 2:**
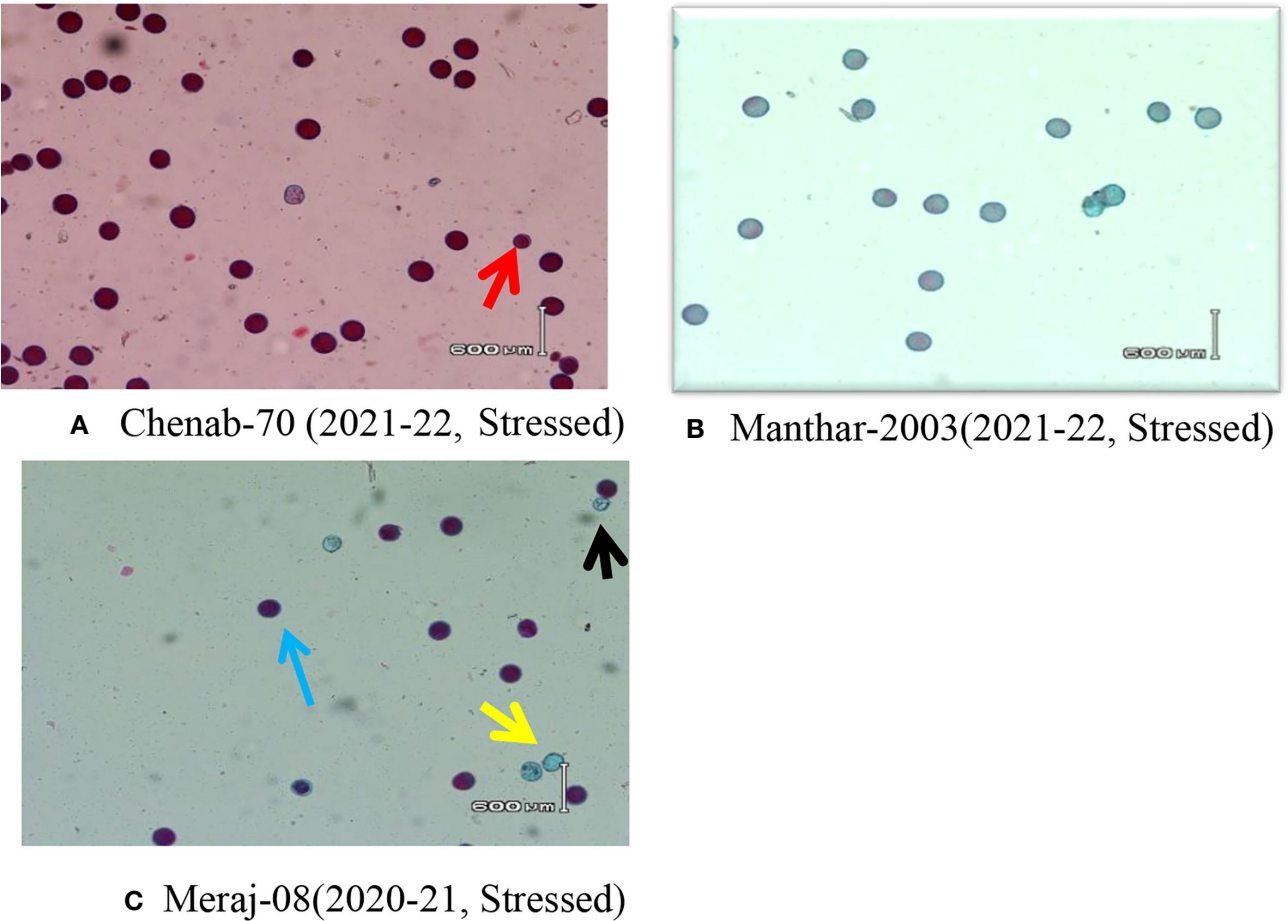
Microphotographs (10×) of wheat lines under stressed conditions during 2020–2021 and 2021–2022. **(A)** Smaller size of viable pollen (red arrow) under stress conditions. **(B)** All sterile/non-viable pollens under stress. **(C)** Meraj-08 stained with Alexander’s stain; the black arrow indicates non-viable pollen grains with a smaller size, the blue arrow indicates viable pollen grains, and the yellow arrow indicates non-viable pollen grains under stress conditions.

**Figure 3:**
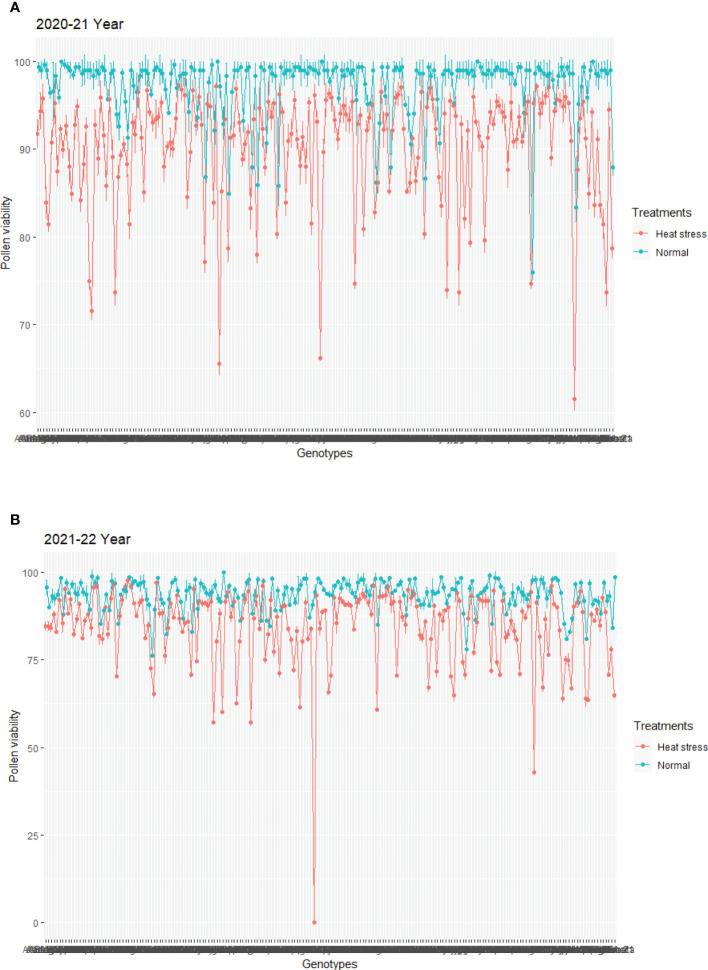
Line plot showing the effect of heat stress on pollen viability in 200 spring wheat genotypes during the period 2020–2021 **(A)** and 2021–2022 **(B)**. The error bars indicate standard deviation (SD) in pollen viability.

Data also showed that pollen viability of some genotypes under heat stress was less affected and had >90% in both years. Moreover, this study showed that viable pollen grains of some genotypes had a smaller size in response to heat stress while some irregular shapes and smaller size were observed in non-viable pollen grains under heat stress (**Figure 2**). The data showed broad genotypic variations for pollen viability under control and heat stress conditions in both years (**Figure 3**). ANOVA showed that genotypes and treatments had a significant effect on pollen viability (**Table 1**). A significant genotype × treatment (G × T) interaction for pollen viability was also observed. The ANOVA results suggested that significant genotypic variation for pollen viability under normal and heat stress conditions could be used to improve pollen viability trait in wheat.

**Table 1:** Analysis of variance (ANOVA) of pollen viability during 2020–2021 and 2021–2022.

Source of variation	df	Sum sq	Mean sq	*F* value	Pr(>*F*)
Treatment	1	40,809	40,809	1126	<2e-16 ***
Year	1	34,196	172	4.74	<2e-16 ***
Genotypes	199	13,860	13,860	382	<2e-16 ***
Treatment × genotypes	199	19,810	100	2.75	<2e-16 ***
Residuals	1,999	72,405	36		

Significance codes: 0 “***”.

The heat susceptibility index is a useful criterion to select heat-tolerant genotypes. The genotypes with *HSI*_*pv*_ < 0.5 were considered as heat tolerant, genotypes with *HSI*_*pv*_ 0.5–0.99 were considered as moderately tolerant, and genotypes with *HSI*_*pv*_ > 1.0 were considered as susceptible. During 2020–2021, 50 genotypes were determined as tolerant, 76 genotypes were determined as moderately tolerant, and 74 genotypes were determined as susceptible. During 2021–2022, 46 genotypes were found to be tolerant, 84 were found to be moderately tolerant, and 70 were found to be susceptible. However, 12 genotypes, viz., Chenab-70, Pari-73, Pak-81, MIH-21, Punjab-76, NIFA-Aman, NUWYT-63, Swabi-1, Nisnan-21, Frontana, Amin-2000, and Pirsabak-2004, were found to be heat tolerant in both periods (**Table 2**).

**Table 2:** Heat-tolerant line on the basis of *HSI*_*pv*_ in the period 2020–2021 and 2021–2022.

S. No.	Names	Year of release	Pedigree	*HSI*_*pv*_ (2020–2021)	*HSI*_*pv*_ (2021–2022)
1	Chenab-70	1970	C271/WI(E)//SON64 PK 146-12A-4A-0A	0.3	0.1
2	Pari-73	1973	CNO67//SN64/ KLRE/3/ 8156 II23584-303M-0Y-11A-1A-1436-OPAK	0.4	0.2
3	Pak-81	1981	KVZ//BUHO//KAL/ BB CM33027-F-15M-500Y-0M-76B-OY-OPAK	0.4	0.3
4	MH-21	2021	WAXWING/4/SNI/TRAP#1/3/ KAUZ*2/TRAP//KAUZ/5/TECUE#1 CMSS06B00468S-0Y-099ZTM-099Y-099M-1W GY-0B	0.2	0.4
5	Punjab-76	1976	NAI60/CB151//S949/ 3/MEXIPAK PK6841-2A-2A-1A-0A	0.03	0.3
6	NIFA-Aman	2016	PRL/2*PASTOR//PBW 343*2/ KUKUNA/3/ ROLY07 29CMSS04B00025T-0TOPY-09922TM-099Y-8W GY-0B	0.2	0.2
7	NUWYT-63	Advanced line		0.4	0.3
8	Swabi-1	2020	ND643/2*WBLL1/4/WHEAR/KUKUNA/3/C80.1/3* BATA VIA//2*WBLL1CM SS08Y00234S-099Y-099M-099NJ-9W GY-0B	0.2	0.2
9	Nisnan-21	2021	(NAC/TH_AC//3*PVN/3/MIRLO/BUC/4/*2PASTOR) /4/HUA234 1T.6T.4T.5T.8T.0T	0.4	0.3
10	Frontana	1940 (Brazilian)	FRONTEIRA/MENTANA	0.4	0.3
11	Amin-2000	2000	PASTOR/OPATA CM 110624-7M -020Y-010M-010SY-010M-0M -0Y	0.4	0.3
12	Pirsabak 2004	2004	KAUZ/STAR	0.2	0.4

**Figure 4:**
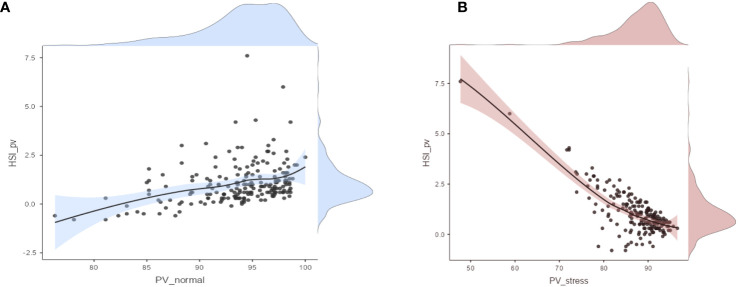
**(A)** Scatter plot between *HSI*_*pv*_ and pollen viability under normal conditions (PV_normal). **(B)** Scatter plot between *HSI*_*pv*_ and pollen viability under stress conditions (PV_stress).

**Figure 5:**
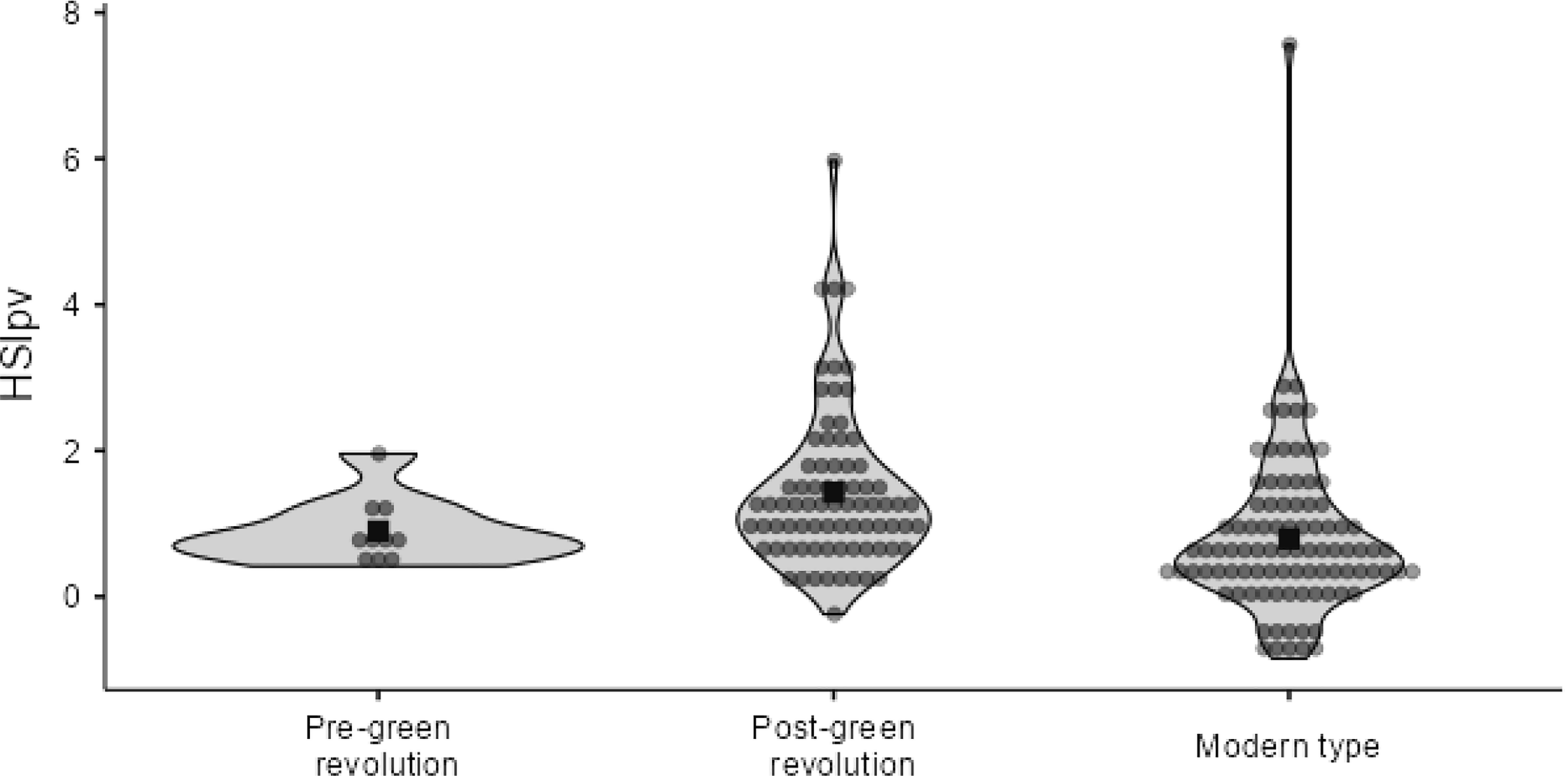
Violin plot showing the trend of *HSI*_*pv*_ across pre-green revolution, post-green revolution, and modern wheat varieties.

To visualize the distribution of data and correlation of pollen viability under normal and heat stress conditions, scatter plots between pollen viability under normal conditions and *HSI*_*pv*_, and pollen viability under heat stress conditions and *HSI*_*pv*_ were constructed. The scatter plot results revealed that pollen viability under normal conditions was positively correlated (*R* = 0.362**) with *HSI*_*pv*_ (**Figure 4A**). Conversely, pollen viability under heat stress conditions was strongly and negatively correlated (*R* = −0.776**) with *HSI*_*pv*_ (**Figure 4B**). These results indicated that under heat stress, genotypes with higher pollen viability could be selected as heat-tolerant genotypes with lower *HSI*_*pv*_. To check the trend of heat tolerance improvement over the periods of wheat breeding history, violin plot was constructed using 2-years average data. The violin plot displayed a trend of improvement in pollen viability-based heat tolerance (*HSI*_*pv*_ < 0.5) over the period of wheat breeding history. Some modern wheat varieties had lower values compared to those grouped in post-green revolution and pre-green revolution categories (**Figure 5**).

**Figure 6:**
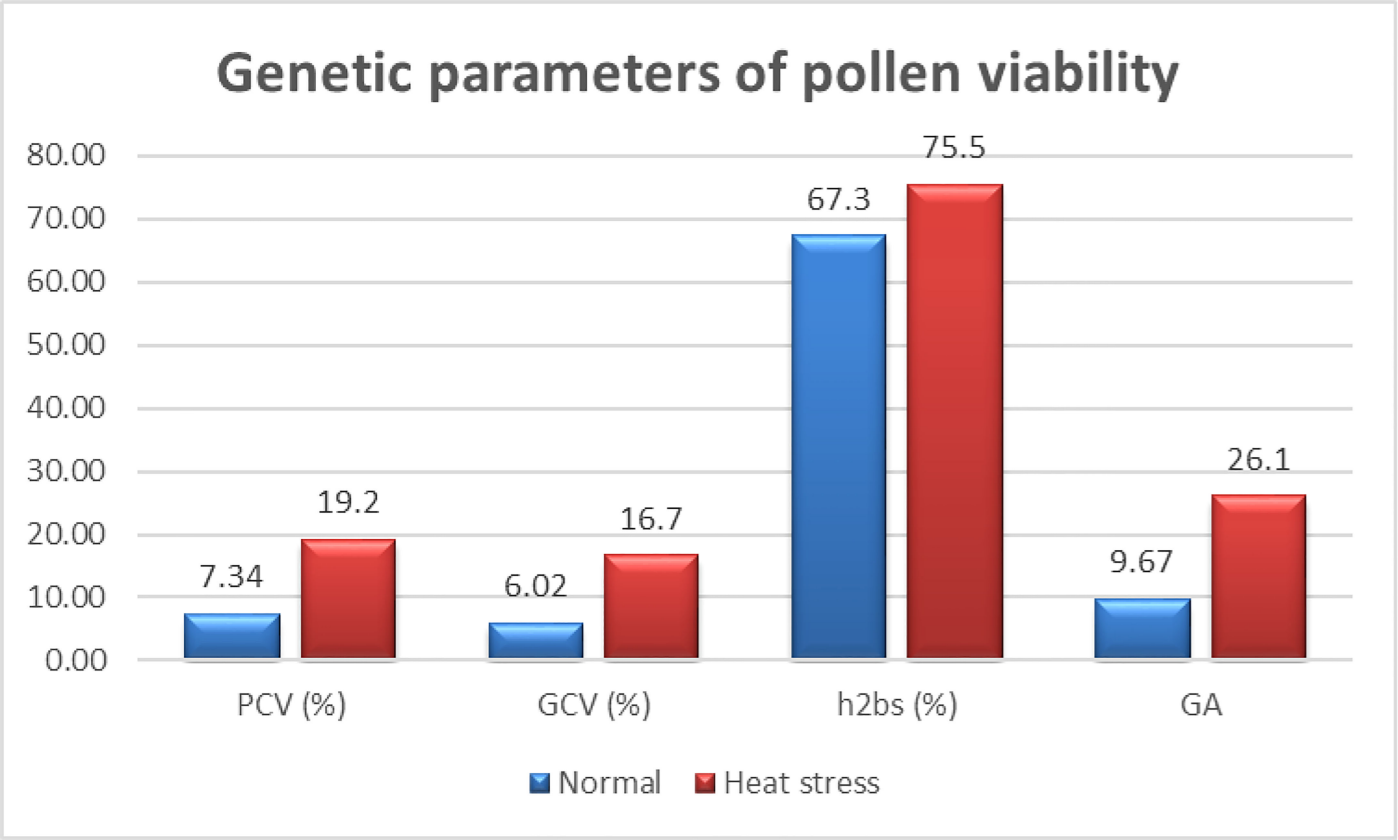
Genetic parameters for pollen viability (%) under normal and heat stress conditions.

To determine the effectiveness of pollen viability in wheat breeding programs, PCV%, GCV%, h^2^_bs_, and GA were estimated. The PCV and GCV provided an insight into the nature of variation for pollen viability in the germplasm. Very minor difference between PCV% and GCV% under control and heat stress conditions indicated that large amount of variation was contributed by genetic components and less by environmental factors (**Figure 6**). The values of PCV% and GCV% under heat stress were higher than normal conditions, suggesting pollen viability as a heat-responsive trait. Heritability estimate (h^2^_bs_) under heat stress conditions (75.5%) was higher than that under normal conditions (67.3%) (**Figure 6**). Besides higher h^2^_bs_ estimate under heat stress, the value of GA for pollen viability under heat stress (26.1) was almost three times higher than the value under normal conditions (9.67).

Taken together, the results of PCV%, GCV%, h^2^_bs_, GA, and scatter plots suggested that pollen viability was largely controlled by heritable genetic components and could be used as a strong selection criterion for heat-tolerant genotypes. The higher values of PCV%, GCV%, h^2^_bs_, and GA under heat stress conditions suggested that pollen viability was highly responsive to heat stress and could be used as a key trait for the selection of heat-tolerant genotypes under elevated temperatures.

## Discussion

Wheat is a heat-sensitive crop (Akter and Rafiqul Islam, 2017) and is susceptible to high temperature during vegetative and reproductive stages (Balla et al., 2019). The optimum temperature for spring wheat during anthesis and grain filling stages ranges from 12 to 22°C (Farooq et al., 2011), and temperatures >30°C are reported to have a negative effect on the wheat yield and seed set (Barnabas et al., 2008). Breeding efforts for temperature tolerance in wheat are hampered due to less information on the heat tolerance mechanism and the unavailability of tolerant wheat genotypes. In such conditions, wheat breeders need genotypes that are highly tolerant against heat stress that support breeding programs for heat stress tolerance.

To determine how pollen viability of wheat responds to terminal heat stress at the reproductive stage, this study was performed to assess the effects of temperature stress on pollen viability of 200 diverse spring wheat genotypes under field conditions. Moreover, selection criteria based on the HSI method were used for identifying heat-tolerant wheat lines among all studied 200 lines of spring wheat under field conditions.

In the present study, heat stress >~30°C was observed during the reproductive stages and during the development of wheat under heat stress conditions that adversely affected pollen formation, fertilization, and seed set in genotypes (Saini et al., 1984; Kaushal et al., 2016). In wheat, pollen formation is the most heat-sensitive stage (SakataHigashitani, 2008). Heat stress-induced pollen sterility in wheat is largely due to irregularities during microsporogenesis (Jager et al., 2008). This high temperature during microsporogenesis decreased the floret fertility and thus caused pollen sterility, low pollen germination, and retarded pollen tube growth, and negatively affected fertilization (Müller and Rieu, 2016).

The results showed that heat stress reduced the pollen viability in both years. This decrease in pollen viability was due to heat stress at the reproductive stage, i.e., flowering stage. This finding is supported by the observation of Browne et al. (2021) that pollen viability and seed set of wheat are greatly reduced under high temperatures, i.e., 30°C, at the flowering stage. Similarly, Aiqing et al. (2018) reported that wheat genotypes that bloomed and flowered in heat stress produced approximately 16% lower seed set compared to those that flowered in the morning; Jager et al. (2008) reported 35% reduction in pollen viability under 30°C in wheat. These findings are in agreement with our results.

In the present study, many genotypes had high pollen viability under normal conditions and low pollen viability under stress conditions in both years. The result showed that during the first year, 2020–2021, pollen viability of genotype Sindhu-16 (76%) under normal conditions and genotypes Meraj-08 (66%), Gulzar-19 (66%), and Ujala-15 (62%) under stress conditions was affected more negatively as compared to other genotypes. However, during the second year, 2021–2022, genotypes Chakwal-86 (76%) and Punjab-96 (78%) under normal conditions and genotypes Janbaz (57%), Ghazi-2019 (57%), Sindhu-16 (43%), and Manthar-2003(0%) under stress conditions were affected and showed reduced pollen viability as compared to other genotypes. The decrease in pollen viability in wheat as a result of high temperature has been determined previously (Wheeler et al., 1996; Prasad et al., 2006; Sharma et al., 2013; Mamrutha et al., 2022).

The result confirmed that some genotypes had high pollen viability >90% under heat stress in both seasons, and there was no effect on pollen shape and size. However, many genotypes showed an irregular shape and a smaller size of non-viable pollens, and some genotypes showed a smaller size of viable pollens under stress. These results are in agreement with some earlier researchers (SainiAspinall, 1982; Saini et al., 1983; Reynolds et al., 2012; Bhardwaj et al., 2017; Nasehzadeh and Ellis, 2017; Mamrutha et al., 2022).

The selection criteria in heat stress is based on various morphological and physiological performances under temperature stress conditions. Heat susceptible indices are considered the best selection criteria for evaluating heat-tolerant genotypes under heat stress conditions (Bhardwaj et al., 2017; Shenoda et al., 2021). The result showed that on the basis of *HSI*_*pv*_ , genotypes were grouped into three classes: highly tolerant, moderate, and susceptible. The heat susceptibility index showed that 12 genotypes, viz., Chenab-70, Pari-73, Pak-81, MIH-21, Punjab-76, NIFA-Aman, NUWYT-63, Swabi-1, Nisnan-21, Frontana, Amin-2000, and Pirsabak-2004, were found to be heat tolerant in both years (**Table 2**). These genotypes showed stability in high pollen viability across the years and could be useful genetic resources. The results confirmed highly significant differences (*P*< 0.01) between the genotypes (G), between treatments (normal and heat stress), and in the genotypes (G) × treatment (T) interaction for pollen viability. These results are in agreement with earlier reports (PrasadDjanaguiraman, 2014; Pimentel et al., 2015; Youldash et al., 2020). The violin plot revealed *HSI*_*pv*_ -based improvement in heat tolerance over a period of time. The decreasing values of *HSI*_*pv*_ in some modern wheat varieties are more likely due to indirect selection for pollen viability under elevated temperatures. Since pollen viability has very strong correlation with grains per spike, some modern varieties with higher pollen viability under heat stress would have been selected on the basis of higher number of grains per spike.

Pollen viability is an important trait for optimal seed setting and enhancing out-crossing potential of a genotype. To our knowledge, genetic estimates (PCV%, GCV%, h^2^_bs_, and GA) for pollen viability are not reported so far. We are the first to report these genetic parameters, which provided an insight that variation in pollen viability is largely genetically controlled and less influenced by the environment. Furthermore, it is responsive to heat stress and have higher h^2^_bs_ and GA values under heat stress. A trait with high h^2^_bs_ estimate along with high GA estimate is considered to be heritable due to additive gene effects, and selection is effective in early generations (Gulnaz et al., 2011). Herein, the values of h^2^_bs_ (75.5%) and GA (26.1) for pollen viability under heat stress are higher than or comparable with most of the quantitative traits inherited due to additive effects (Gulnaz et al., 2011; Shivom et al., 2020). Based on these comparisons, pollen viability can be considered as inherited traits with additive effects and can be selected in early generations to improve genetic populations for higher pollen viability under heat stress conditions. Recently, a tightly linked marker [Excalibur_rep_c109881_701 (7A)] to pollen viability was reported to contribute 19.35% of the observed phenotypic variation (El Hanafi et al., 2021). The genome-wide association study for pollen viability traits could be rewarding in terms of discovering new marker trait associations.

## Conclusion

The present study determined that heat stress negatively affected pollen viability of most of the genotypes and also showed broad genotypic diversity for this trait. Some highly tolerant wheat lines were found on the basis of the heat susceptibility index, which are useful for future breeding programs. Few modern spring wheat varieties (released after 2001) were heat tolerant across the years, emphasizing the focus on pollen viability trait in present-day wheat breeding programs. The present findings showed that pollen viability is an important trait for screening heat-tolerant wheat genotypes. Moreover, pollen viability-based selection for heat-tolerant lines in early generation of a segregating population will be rewarding in selecting truly heat-tolerant lines.

## Data availability statement

The original contributions presented in the study are included in the article/**Supplementary Material**. Further inquiries can be directed to the corresponding author.

## Author contributions

IK, executed the experiment, recorded and analyzed data and wrote the first draft; JW, critically reviewed and improved the draft as co-supervisor; MS, conceived the idea, supervised the research work and improved the student draft as her PhD supervisor. All authors contributed to the article and approved the submitted version.

## Funding

This work was supported by the Shandong Provincial Natural Science Foundation project (ZR2021ZD31).

## Acknowledgments

The authors would like to thank Dr. Awais Rasheed (QAU), Dr. Armghan Shahzad (NIGAB, NARC). and Dr. Zahid Mahmood (CSI, NARC) for their aid in providing seed, planting, and managing field experiments at NARC. The authors would also like to thank Dr. Muhammad Umer (CUI) and Dr. Inam Afzal for their support in microscopy work.

## Conflict of interest

The authors declare that the research was conducted in the absence of any commercial or financial relationships that could be construed as a potential conflict of interest.

## Publisher’s note

All claims expressed in this article are solely those of the authors and do not necessarily represent those of their affiliated organizations, or those of the publisher, the editors and the reviewers. Any product that may be evaluated in this article, or claim that may be made by its manufacturer, is not guaranteed or endorsed by the publisher.
