# Supplementary material for: Pollen viability-based heat susceptibility index (HSIpv): A useful selection criterion for heat-tolerant genotypes in wheat
Source: Front Plant Sci. 2022 Dec 1;13:1064569. doi: 10.3389/fpls.2022.1064569 (PMC9751600; doi:10.3389/fpls.2022.1064569)
Supplement: Supplementary file 1 [file DataSheet_1.docx]

| **Table S1: Pollen viability(%) data under normal and heat stress condition fpr the year 2020-21** | | | | |
| --- | --- | --- | --- | --- |
|  | |  | Pollen viability (%) 2020-21 | |
| Genotypes | | Name | Normal | Heat stress |
| 1 | | Chenab-70 | 96.3 | 94.2 |
| 2 | | T-9 | 95.2 | 94.3 |
| 3 | | T-11 | 97.9 | 89.0 |
| 4 | | Sarab-92 | 98.6 | 94.3 |
| 5 | | C-518 | 97.3 | 93.0 |
| 6 | | C-591 | 96.6 | 91.7 |
| 7 | | C-228 | 98.7 | 89.3 |
| 8 | | C-217 | 92.6 | 86.8 |
| 9 | | C-271 | 91.3 | 88.3 |
| 10 | | Saddiq-21 | 98.6 | 79.7 |
| 11 | | Pari-73 | 94.0 | 91.2 |
| 12 | | Pak-81 | 95.1 | 92.3 |
| 13 | | C-250 | 95.5 | 90.6 |
| 14 | | Dilkash | 96.8 | 88.0 |
| 15 | | MIH-21 | 97.7 | 96.4 |
| 16 | | Nawab-21 | 99.1 | 93.9 |
| 17 | | Punjab-76 | 95.3 | 95.0 |
| 18 | | Anaj-2017 | 95.9 | 87.5 |
| 19 | | Fakhr-e-Bhakkar | 94.2 | 84.6 |
| 20 | | Barani-17 | 99.0 | 75.0 |
| 21 | | Bhakkar star | 95.7 | 85.8 |
| 22 | | Markaz-2019 | 99.1 | 81.6 |
| 23 | | NIFA- Awaz | 95.2 | 92.1 |
| 24 | | Faheem-19 | 97.0 | 93.5 |
| 25 | | Ghazi-2019 | 97.7 | 95.1 |
| 26 | | NIA-Zakheera | 97.4 | 80.9 |
| 27 | | NIA-Shaheen | 95.6 | 74.7 |
| 28 | | Akber-19 | 96.6 | 90.8 |
| 29 | | Gulzar-19 | 97.2 | 65.6 |
| 30 | | Pirsbak-19 | 98.5 | 83.5 |
| 31 | | Rawal-87 | 100.0 | 93.1 |
| 32 | | Anmol-91 | 100.0 | 92.3 |
| 33 | | Dirk | 94.2 | 90.3 |
| 34 | | Morocco | 95.8 | 91.1 |
| 35 | | agaaz | 96.4 | 81.5 |
| 36 | | Khoshn | 90.7 | 88.0 |
| 37 | | AZRC | 98.4 | 84.9 |
| 38 | | Umeed-e-Khas | 97.1 | 93.5 |
| 39 | | WL 711 | 100.0 | 94.2 |
| 40 | | Pirsbak-13 | 95.7 | 92.3 |
| 41 | | Hamal-Faqir | 92.9 | 85.2 |
| 42 | | Pakistan-2013 | 90.6 | 86.2 |
| 43 | | NIFA-Lilma | 93.0 | 86.2 |
| 44 | | Galaxy-2013 | 86.8 | 77.2 |
| 45 | | Shalakot-13 | 94.2 | 90.9 |
| 46 | | Umeed-2014 | 95.2 | 87.7 |
| 47 | | Pakhtunkhwa-15 | 94.0 | 85.2 |
| 48 | | Pirsbak-15 | 90.7 | 86.8 |
| 49 | | Ujala-15 | 83.4 | 61.5 |
| 50 | | IBGE-Ghaneemat | 84.9 | 78.7 |
| 51 | | NIFA-Insaf | 86.2 | 82.8 |
| 52 | | Sindhu-16 | 76.0 | 74.7 |
| 53 | | Zincol | 88.0 | 78.7 |
| 54 | | Boroloug-16 | 93.9 | 73.7 |
| 55 | | NIFA-Aman | 95.2 | 93.6 |
| 56 | | Gold-16 | 92.1 | 83.9 |
| 57 | | Johar-16 | 88.0 | 83.2 |
| 58 | | Ihsan-16 | 96.5 | 91.3 |
| 59 | | Fatehjang-16 | 93.6 | 92.7 |
| 60 | | NN-Gandum1 | 96.1 | 92.2 |
| 61 | | Wardan-17 | 95.9 | 91.2 |
| 62 | | Pasina17 | 86.7 | 80.3 |
| 63 | | Khaista-17 | 85.9 | 78.0 |
| 64 | | Israr Shaheed | 93.2 | 88.8 |
| 65 | | Shahid-17 | 97.5 | 90.9 |
| 66 | | Kohat-17 | 85.8 | 80.3 |
| 67 | | NUWYT-63 | 88.0 | 85.2 |
| 68 | | Meraj-08 | 100.0 | 66.2 |
| 69 | | C-273 | 99.0 | 81.4 |
| 70 | | Mexipak 65 | 99.0 | 89.6 |
| 71 | | Khushal-69 | 99.3 | 95.5 |
| 72 | | Pirsbaq-21 | 99.0 | 74.0 |
| 73 | | Barani-70 | 98.3 | 71.6 |
| 74 | | Blue silver | 99.0 | 89.1 |
| 75 | | SA-42 | 98.7 | 90.3 |
| 76 | | Lylpur-73 | 99.0 | 88.1 |
| 77 | | Erra | 99.7 | 90.0 |
| 78 | | Parula-73 | 99.0 | 86.4 |
| 79 | | Yacora | 98.7 | 83.7 |
| 80 | | LU-26 | 99.3 | 91.1 |
| 81 | | Abbaser-21 | 99.0 | 83.9 |
| 82 | | Zargoun-21 | 98.7 | 73.7 |
| 83 | | Jauhar-78 | 99.3 | 92.0 |
| 84 | | Zarghoon | 99.0 | 81.4 |
| 85 | | SWB-1 | 98.7 | 97.1 |
| 86 | | Punjab-81 | 99.0 | 93.8 |
| 87 | | Sind-81 | 99.0 | 95.5 |
| 88 | | Faisalabad 83 | 98.7 | 96.8 |
| 89 | | Kohinoor 83 | 99.3 | 96.2 |
| 90 | | Barani-83 | 99.0 | 92.8 |
| 91 | | Faisalabad 85 | 98.7 | 96.2 |
| 92 | | Sarsabz | 99.0 | 92.8 |
| 93 | | Tandojam 83 | 99.3 | 95.5 |
| 94 | | Pirsbak-85 | 99.0 | 94.1 |
| 95 | | Punjab-85 | 99.0 | 73.7 |
| 96 | | Wdank-85 | 99.0 | 84.9 |
| 97 | | Chakwal 86 | 99.0 | 85.1 |
| 98 | | MA-2021 | 99.0 | 91.4 |
| 99 | | Khyber 87 | 98.7 | 93.7 |
| 100 | | Sutluj-86 | 98.3 | 96.0 |
| 101 | | Shalimar-88 | 99.0 | 94.1 |
| 102 | | Zardana | 99.0 | 83.7 |
| 103 | | Mehran-89 | 98.7 | 93.1 |
| 104 | | Rohtas 90 | 99.3 | 91.5 |
| 105 | | Inqilab 91 | 99.0 | 96.9 |
| 106 | | Pasban 90 | 98.7 | 96.5 |
| 107 | | Soghat-90 | 99.3 | 97.2 |
| 108 | | Bakhtawar 92 | 99.0 | 92.6 |
| 109 | | Kaghan 93 | 98.3 | 93.4 |
| 110 | | Nisnan | 99.3 | 96.5 |
| 111 | | Shaheen 94 | 98.7 | 95.4 |
| 112 | | Parwaz 94 | 98.7 | 89.0 |
| 113 | | Shahkar 95 | 99.3 | 93.7 |
| 114 | | Kohsar 95 | 99.0 | 83.9 |
| 115 | | Kirin 95 | 98.3 | 95.3 |
| 116 | | Nowshera 96 | 99.3 | 93.9 |
| 117 | | Punjab 96 | 99.0 | 82.1 |
| 118 | | Suleman 96 | 98.7 | 95.3 |
| 119 | | Abadgar 93 | 99.7 | 95.8 |
| 120 | | Frontana | 99.0 | 96.0 |
| 121 | | Tatara | 98.3 | 94.9 |
| 122 | | AZRI-96 | 99.3 | 92.8 |
| 123 | | Punjab-96 | 99.0 | 92.9 |
| 124 | | Bahawalpur-97 | 98.7 | 84.2 |
| 125 | | NUWYT-64 | 99.0 | 92.1 |
| 126 | | MH-97 | 99.0 | 95.6 |
| 127 | | Kohistan 97 | 98.7 | 94.2 |
| 128 | | Fakhr-e-Sarhad | 99.3 | 89.6 |
| 129 | | Chakwal-97 | 99.0 | 91.3 |
| 130 | | Durum-97 | 98.7 | 90.8 |
| 131 | | Darawar-97 | 99.3 | 93.4 |
| 132 | | Daman-98 | 99.0 | 93.0 |
| 133 | | Dera-98 | 99.0 | 93.7 |
| 134 | | Ghaznavi | 99.7 | 94.9 |
| 135 | | Zarlashata | 99.0 | 94.5 |
| 136 | | Magalla-99 | 98.7 | 88.0 |
| 137 | | Bahawalpur-2000 | 99.3 | 94.9 |
| 138 | | Marvi-2000 | 99.0 | 96.2 |
| 139 | | Takbeer | 98.3 | 95.7 |
| 140 | | Iqbal 2000 | 99.3 | 93.0 |
| 141 | | Auqab-2000 | 99.0 | 88.0 |
| 142 | | Amin-2000 | 98.3 | 95.2 |
| 143 | | NUWYT-65 | 99.3 | 95.9 |
| 144 | | Saleem 2000 | 99.0 | 91.3 |
| 145 | | Punjnad-1 | 98.7 | 92.1 |
| 146 | | Khatakwal | 99.3 | 94.7 |
| 147 | | Raj | 99.0 | 79.3 |
| 148 | | Chenab-2000 | 98.7 | 96.7 |
| 149 | | Wafaq-01 | 99.3 | 95.4 |
| 150 | | SH-2002 | 99.0 | 93.7 |
| 151 | | Bahkhar-2002 | 99.0 | 88.3 |
| 152 | | AS-2002 | 99.7 | 90.0 |
| 153 | | GA-2002 | 99.0 | 92.8 |
| 154 | | Ufaq | 98.7 | 91.0 |
| 155 | | Manthar-2003 | 99.3 | 95.3 |
| 156 | | KT-2000 | 99.0 | 91.0 |
| 157 | | Pirsabak 2004 | 98.3 | 97.0 |
| 158 | | NUWYT-66 | 99.0 | 96.3 |
| 159 | | Bhittai | 98.7 | 95.7 |
| 160 | | Zam-04 | 98.3 | 91.1 |
| 161 | | TD-1 | 99.3 | 95.9 |
| 162 | | Pirsabak 2005 | 99.0 | 95.7 |
| 163 | | Rashkoh-2005 | 98.3 | 96.0 |
| 164 | | Moomal 2002 | 99.3 | 93.3 |
| 165 | | Sehar -2006 | 99.0 | 95.0 |
| 166 | | Shafaq -2006 | 98.7 | 87.7 |
| 167 | | Saussi | 99.3 | 95.5 |
| 168 | | Fareed-06 | 99.0 | 96.7 |
| 169 | | SKD-1 | 98.7 | 95.3 |
| 170 | | Imdad-05 | 99.0 | 91.6 |
| 171 | | Khirman | 99.0 | 92.4 |
| 172 | | Chakwal-50 | 98.3 | 96.5 |
| 173 | | Lasani-08 | 99.3 | 95.6 |
| 174 | | Subhani | 99.0 | 94.1 |
| 175 | | Faisalabad-08 | 98.3 | 97.2 |
| 176 | | Bathoor-08 | 99.3 | 95.9 |
| 177 | | NUWYT-67 | 99.3 | 97.0 |
| 178 | | Hashim-08 | 98.3 | 93.4 |
| 179 | | Pirsabak-2008 | 99.3 | 94.8 |
| 180 | | NARC-2009 | 99.0 | 94.1 |
| 181 | | BARS-2009 | 98.7 | 89.0 |
| 182 | | AARI-2010 | 99.3 | 91.8 |
| 183 | | NIFA-Barsat10 | 99.0 | 95.1 |
| 184 | | NIA-Amber | 98.7 | 93.3 |
| 185 | | NIA-Sunehri | 99.3 | 93.9 |
| 186 | | Janbaz | 99.0 | 90.6 |
| 187 | | Tijban-10 | 98.7 | 95.2 |
| 188 | | Atta Habib | 99.3 | 92.7 |
| 189 | | Seren | 99.0 | 94.3 |
| 190 | | KT-2010 | 98.3 | 91.8 |
| 191 | | Aas-2011 | 99.0 | 94.3 |
| 192 | | Gomal-08 | 100.0 | 97.2 |
| 193 | | Punjab-2011 | 98.7 | 95.5 |
| 194 | | Millat-2011 | 99.0 | 95.9 |
| 195 | | Dharrabi-11 | 98.7 | 95.4 |
| 196 | | NARC-2011 | 98.3 | 95.0 |
| 197 | | NIA-Sunder | 99.3 | 92.9 |
| 198 | | NIA-Sarang | 99.0 | 95.5 |
| 199 | | Benazir-13 | 99.0 | 91.6 |
| 200 | | Shahkar-13 | 99.3 | 91.1 |

| **Table S2: Pollen viability(%) data under normal and heat stress condition fpr the year 2021-22** | | | |
| --- | --- | --- | --- |
|  |  | Pollen viability (%) 2021-22 | |
| Genotypes | Names | Normal | Heat stress |
| 1 | Chenab-70 | 98.5 | 97.1 |
| 2 | T-9 | 97.9 | 90.0 |
| 3 | T-11 | 98.5 | 91.0 |
| 4 | Sarab-92 | 98.6 | 94.8 |
| 5 | C-518 | 96.8 | 91.3 |
| 6 | C-591 | 97.3 | 92.2 |
| 7 | C-228 | 98.6 | 97.7 |
| 8 | C-217 | 94.6 | 96.0 |
| 9 | C-271 | 97.5 | 91.1 |
| 10 | Saddiq-21 | 99.2 | 92.0 |
| 11 | Pari-73 | 92.1 | 90.3 |
| 12 | Pak-81 | 97.9 | 95.0 |
| 13 | C-250 | 96.9 | 96.0 |
| 14 | Dilkash | 97.2 | 91.3 |
| 15 | MIH-21 | 96.2 | 92.8 |
| 16 | Nawab-21 | 94.2 | 90.6 |
| 17 | Punjab-76 | 97.0 | 94.2 |
| 18 | Anaj-2017 | 97.1 | 95.3 |
| 19 | Fakhr-e-Bhakkar | 98.1 | 95.2 |
| 20 | Barani-17 | 98.4 | 96.1 |
| 21 | Bhakkar star | 96.9 | 96.7 |
| 22 | Markaz-2019 | 98.2 | 93.5 |
| 23 | NIFA- Awaz | 96.1 | 88.0 |
| 24 | Faheem-19 | 86.8 | 83.1 |
| 25 | Ghazi-2019 | 96.5 | 57.0 |
| 26 | NIA-Zakheera | 93.5 | 91.4 |
| 27 | NIA-Shaheen | 98.0 | 92.3 |
| 28 | Akber-19 | 98.5 | 92.1 |
| 29 | Gulzar-19 | 93.3 | 91.7 |
| 30 | Pirsbak-19 | 91.2 | 89.2 |
| 31 | Rawal-87 | 94.2 | 92.3 |
| 32 | Anmol-91 | 94.2 | 88.3 |
| 33 | Dirk | 98.1 | 87.2 |
| 34 | Morocco | 98.2 | 91.8 |
| 35 | agaaz | 93.8 | 83.1 |
| 36 | Khoshn | 98.2 | 92.1 |
| 37 | AZRC | 97.1 | 90.9 |
| 38 | Umeed-e-Khas | 92.0 | 88.8 |
| 39 | WL 711 | 92.1 | 86.1 |
| 40 | Pirsbak-13 | 98.8 | 88.0 |
| 41 | Hamal-Faqir | 96.3 | 94.3 |
| 42 | Pakistan-2013 | 98.2 | 94.0 |
| 43 | NIFA-Lilma | 97.3 | 93.3 |
| 44 | Galaxy-2013 | 94.4 | 91.7 |
| 45 | Shalakot-13 | 92.3 | 87.1 |
| 46 | Umeed-2014 | 97.1 | 94.6 |
| 47 | Pakhtunkhwa-15 | 93.8 | 92.7 |
| 48 | Pirsbak-15 | 95.7 | 86.3 |
| 49 | Ujala-15 | 91.5 | 91.3 |
| 50 | IBGE-Ghaneemat | 95.0 | 91.6 |
| 51 | NIFA-Insaf | 98.2 | 93.0 |
| 52 | Sindhu-16 | 97.9 | 42.9 |
| 53 | Zincol | 98.6 | 64.8 |
| 54 | Boroloug-16 | 95.0 | 92.1 |
| 55 | NIFA-Aman | 98.3 | 96.1 |
| 56 | Gold-16 | 91.8 | 88.2 |
| 57 | Johar-16 | 97.9 | 93.2 |
| 58 | Ihsan-16 | 92.8 | 62.5 |
| 59 | Fatehjang-16 | 95.7 | 91.1 |
| 60 | NN-Gandum1 | 98.5 | 96.0 |
| 61 | Wardan-17 | 97.0 | 63.5 |
| 62 | Pasina17 | 90.6 | 67.1 |
| 63 | Khaista-17 | 97.6 | 96.2 |
| 64 | Israr Shaheed | 98.1 | 94.5 |
| 65 | Shahid-17 | 95.5 | 80.7 |
| 66 | Kohat-17 | 93.6 | 90.3 |
| 67 | NUWYT-63 | 94.9 | 91.7 |
| 68 | Meraj-08 | 94.0 | 89.2 |
| 69 | C-273 | 96.4 | 87.7 |
| 70 | Mexipak 65 | 93.6 | 65.9 |
| 71 | Khushal-69 | 89.2 | 77.3 |
| 72 | Pirsbaq-21 | 95.3 | 70.3 |
| 73 | Barani-70 | 85.2 | 81.9 |
| 74 | Blue silver | 93.6 | 87.6 |
| 75 | SA-42 | 95.9 | 92.0 |
| 76 | Lylpur-73 | 98.3 | 91.3 |
| 77 | Erra | 91.6 | 87.0 |
| 78 | Parula-73 | 90.7 | 82.1 |
| 79 | Yacora | 91.8 | 89.3 |
| 80 | LU-26 | 98.3 | 80.3 |
| 81 | Abbaser-21 | 92.5 | 88.0 |
| 82 | Zargoun-21 | 93.4 | 70.8 |
| 83 | Jauhar-78 | 93.5 | 86.8 |
| 84 | Zarghoon | 91.9 | 88.7 |
| 85 | SWB-1 | 98.0 | 96.1 |
| 86 | Punjab-81 | 98.5 | 91.8 |
| 87 | Sind-81 | 97.9 | 94.4 |
| 88 | Faisalabad 83 | 95.8 | 86.1 |
| 89 | Kohinoor 83 | 95.9 | 90.6 |
| 90 | Barani-83 | 89.1 | 81.0 |
| 91 | Faisalabad 85 | 83.1 | 70.8 |
| 92 | Sarsabz | 98.2 | 74.3 |
| 93 | Tandojam 83 | 85.2 | 63.9 |
| 94 | Pirsbak-85 | 93.4 | 90.0 |
| 95 | Punjab-85 | 88.3 | 74.3 |
| 96 | Wdank-85 | 91.0 | 88.0 |
| 97 | Chakwal 86 | 76.3 | 72.6 |
| 98 | MA-2021 | 87.2 | 80.9 |
| 99 | Khyber 87 | 94.9 | 87.3 |
| 100 | Sutluj-86 | 94.6 | 76.5 |
| 101 | Shalimar-88 | 93.8 | 90.3 |
| 102 | Zardana | 97.1 | 92.8 |
| 103 | Mehran-89 | 95.1 | 88.8 |
| 104 | Rohtas 90 | 94.9 | 91.8 |
| 105 | Inqilab 91 | 93.4 | 86.8 |
| 106 | Pasban 90 | 94.5 | 85.9 |
| 107 | Soghat-90 | 98.1 | 81.6 |
| 108 | Bakhtawar 92 | 97.1 | 95.7 |
| 109 | Kaghan 93 | 86.2 | 84.0 |
| 110 | Nisnan | 95.8 | 93.4 |
| 111 | Shaheen 94 | 90.8 | 83.2 |
| 112 | Parwaz 94 | 91.0 | 81.3 |
| 113 | Shahkar 95 | 93.4 | 89.0 |
| 114 | Kohsar 95 | 95.3 | 80.8 |
| 115 | Kirin 95 | 89.3 | 71.2 |
| 116 | Nowshera 96 | 94.2 | 91.0 |
| 117 | Punjab 96 | 95.5 | 87.0 |
| 118 | Suleman 96 | 92.7 | 86.6 |
| 119 | Abadgar 93 | 93.3 | 84.2 |
| 120 | Frontana | 94.2 | 91.2 |
| 121 | Tatara | 81.1 | 75.0 |
| 122 | AZRI-96 | 94.4 | 81.3 |
| 123 | Punjab-96 | 78.1 | 70.7 |
| 124 | Bahawalpur-97 | 89.3 | 88.0 |
| 125 | NUWYT-64 | 96.4 | 70.5 |
| 126 | MH-97 | 93.4 | 70.5 |
| 127 | Kohistan 97 | 90.8 | 83.9 |
| 128 | Fakhr-e-Sarhad | 89.7 | 74.7 |
| 129 | Chakwal-97 | 90.7 | 84.9 |
| 130 | Durum-97 | 94.9 | 93.4 |
| 131 | Darawar-97 | 91.3 | 88.6 |
| 132 | Daman-98 | 92.3 | 88.2 |
| 133 | Dera-98 | 82.3 | 76.3 |
| 134 | Ghaznavi | 92.7 | 80.3 |
| 135 | Zarlashata | 84.1 | 78.0 |
| 136 | Magalla-99 | 90.8 | 83.9 |
| 137 | Bahawalpur-2000 | 93.6 | 86.2 |
| 138 | Marvi-2000 | 97.2 | 84.0 |
| 139 | Takbeer | 94.3 | 83.4 |
| 140 | Iqbal 2000 | 97.0 | 92.4 |
| 141 | Auqab-2000 | 93.6 | 86.6 |
| 142 | Amin-2000 | 88.1 | 85.5 |
| 143 | NUWYT-65 | 97.2 | 91.7 |
| 144 | Saleem 2000 | 85.2 | 71.9 |
| 145 | Punjnad-1 | 97.6 | 93.4 |
| 146 | Khatakwal | 86.1 | 75.0 |
| 147 | Raj | 86.5 | 77.1 |
| 148 | Chenab-2000 | 89.3 | 65.2 |
| 149 | Wafaq-01 | 81.1 | 63.9 |
| 150 | SH-2002 | 91.0 | 82.4 |
| 151 | Bahkhar-2002 | 99.0 | 84.2 |
| 152 | AS-2002 | 96.6 | 92.3 |
| 153 | GA-2002 | 93.7 | 90.6 |
| 154 | Ufaq | 96.7 | 90.3 |
| 155 | Manthar-2003 | 94.5 | 0.0 |
| 156 | KT-2000 | 91.5 | 72.2 |
| 157 | Pirsabak 2004 | 94.0 | 90.6 |
| 158 | NUWYT-66 | 93.6 | 87.3 |
| 159 | Bhittai | 85.2 | 70.3 |
| 160 | Zam-04 | 88.3 | 84.8 |
| 161 | TD-1 | 83.1 | 75.0 |
| 162 | Pirsabak 2005 | 94.5 | 71.6 |
| 163 | Rashkoh-2005 | 93.3 | 85.8 |
| 164 | Moomal 2002 | 95.5 | 92.6 |
| 165 | Sehar -2006 | 94.0 | 91.9 |
| 166 | Shafaq -2006 | 94.2 | 86.3 |
| 167 | Saussi | 96.0 | 70.8 |
| 168 | Fareed-06 | 96.9 | 92.0 |
| 169 | SKD-1 | 93.0 | 91.4 |
| 170 | Imdad-05 | 86.3 | 80.3 |
| 171 | Khirman | 84.7 | 82.4 |
| 172 | Chakwal-50 | 92.3 | 81.3 |
| 173 | Lasani-08 | 96.5 | 61.5 |
| 174 | Subhani | 97.0 | 67.3 |
| 175 | Faisalabad-08 | 94.8 | 85.6 |
| 176 | Bathoor-08 | 89.2 | 82.4 |
| 177 | NUWYT-67 | 87.7 | 85.1 |
| 178 | Hashim-08 | 90.5 | 87.3 |
| 179 | Pirsabak-2008 | 94.7 | 80.9 |
| 180 | NARC-2009 | 96.6 | 90.7 |
| 181 | BARS-2009 | 93.8 | 90.0 |
| 182 | AARI-2010 | 95.8 | 84.6 |
| 183 | NIFA-Barsat10 | 85.0 | 60.8 |
| 184 | NIA-Amber | 93.0 | 83.7 |
| 185 | NIA-Sunehri | 94.6 | 93.0 |
| 186 | Janbaz | 88.3 | 57.1 |
| 187 | Tijban-10 | 86.8 | 66.9 |
| 188 | Atta Habib | 94.2 | 82.4 |
| 189 | Seren | 88.1 | 81.4 |
| 190 | KT-2010 | 95.0 | 83.2 |
| 191 | Aas-2011 | 90.1 | 84.6 |
| 192 | Gomal-08 | 100.0 | 60.0 |
| 193 | Punjab-2011 | 95.1 | 64.9 |
| 194 | Millat-2011 | 97.4 | 85.6 |
| 195 | Dharrabi-11 | 92.3 | 84.2 |
| 196 | NARC-2011 | 96.4 | 91.0 |
| 197 | NIA-Sunder | 98.0 | 93.9 |
| 198 | NIA-Sarang | 97.1 | 91.5 |
| 199 | Benazir-13 | 97.6 | 92.5 |
| 200 | Shahkar-13 | 94.4 | 71.0 |

| Table S3: Descriptive Statistics of the two years pollen viability data under normal and heat stress conditions | | | | | | | | | | | | | |
| --- | --- | --- | --- | --- | --- | --- | --- | --- | --- | --- | --- | --- | --- |
|  | | **PV_normal Year1** | | **PV_stress Year 1** | | **HSI_pv Year1** | | **PV_normal Year 2** | | **PV_stress Year 2** | | **HSI_pv Year 2** | |
| N |  | 200 |  | 200 |  | 200 |  | 200 |  | 200 |  | 200 |  |
| Missing |  | 0 |  | 0 |  | 0 |  | 0 |  | 0 |  | 0 |  |
| Mean |  | 97.4 |  | 90.2 |  | 1.000 |  | 93.6 |  | 84.3 |  | 1.00 |  |
| Median |  | 98.7 |  | 92.3 |  | 0.736 |  | 94.4 |  | 87.3 |  | 0.651 |  |
| Standard deviation |  | 3.54 |  | 6.60 |  | 0.806 |  | 4.36 |  | 11.4 |  | 1.13 |  |
| Minimum |  | 76.0 |  | 61.5 |  | 0.0400 |  | 76.3 |  | 0.00 |  | -0.148 |  |
| Maximum |  | 100 |  | 97.2 |  | 4.59 |  | 100 |  | 97.7 |  | 10.0 |  |
|  | | | | | | | | | | | | | |

| **Table S4: Descriptive Statistics of the categorical two years pollen viability data under normal and heat stress conditions** | | | | | | | |
| --- | --- | --- | --- | --- | --- | --- | --- |
|  | | **Catagory** | | **HSI_pv 1** | | **HSI_pv 2** | |
| N |  | Pre-green revolution |  | 10 |  | 10 |  |
|  |  | Post-green revolution |  | 77 |  | 77 |  |
|  |  | Modern type |  | 93 |  | 93 |  |
| Missing |  | Pre-green revolution |  | 0 |  | 0 |  |
|  |  | Post-green revolution |  | 0 |  | 0 |  |
|  |  | Modern type |  | 0 |  | 0 |  |
| Mean |  | Pre-green revolution |  | 0.874 |  | 0.452 |  |
|  |  | Post-green revolution |  | 1.30 |  | 0.838 |  |
|  |  | Modern type |  | 0.823 |  | 1.10 |  |
| Median |  | Pre-green revolution |  | 0.641 |  | 0.547 |  |
|  |  | Post-green revolution |  | 0.999 |  | 0.482 |  |
|  |  | Modern type |  | 0.676 |  | 0.762 |  |
| Standard deviation |  | Pre-green revolution |  | 0.714 |  | 0.326 |  |
|  |  | Post-green revolution |  | 1.03 |  | 1.04 |  |
|  |  | Modern type |  | 0.576 |  | 1.20 |  |
| Minimum |  | Pre-green revolution |  | 0.130 |  | -0.148 |  |
|  |  | Post-green revolution |  | 0.0400 |  | 0.0170 |  |
|  |  | Modern type |  | 0.151 |  | 0.137 |  |
| Maximum |  | Pre-green revolution |  | 2.61 |  | 0.805 |  |
|  |  | Post-green revolution |  | 4.59 |  | 5.64 |  |
|  |  | Modern type |  | 3.48 |  | 10.0 |  |

| **Table S5: Descriptive Statistics of the categorical mean HSIpv of the two years pollen viability data under normal and heat stress conditions** | | | | | |
| --- | --- | --- | --- | --- | --- |
|  | | **Catagory** | | **HSIpv** | |
| N |  | Pre-green revolution |  | 10 |  |
|  |  | Post-green revolution |  | 77 |  |
|  |  | Modern type |  | 93 |  |
| Missing |  | Pre-green revolution |  | 0 |  |
|  |  | Post-green revolution |  | 0 |  |
|  |  | Modern type |  | 0 |  |
| Mean |  | Pre-green revolution |  | 0.899 |  |
|  |  | Post-green revolution |  | 1.43 |  |
|  |  | Modern type |  | 0.793 |  |
| Median |  | Pre-green revolution |  | 0.776 |  |
|  |  | Post-green revolution |  | 1.15 |  |
|  |  | Modern type |  | 0.557 |  |
| Standard deviation |  | Pre-green revolution |  | 0.458 |  |
|  |  | Post-green revolution |  | 1.08 |  |
|  |  | Modern type |  | 1.10 |  |
| Minimum |  | Pre-green revolution |  | 0.417 |  |
|  |  | Post-green revolution |  | -0.243 |  |
|  |  | Modern type |  | -0.848 |  |
| Maximum |  | Pre-green revolution |  | 1.96 |  |
|  |  | Post-green revolution |  | 5.97 |  |
|  |  | Modern type |  | 7.56 |  |
|  | | | | | |

|  |
| --- |

**B**

**A**
